# Weight misperception among Chinese children and adolescents: evidence from the repeated China Health and Nutrition Survey

**DOI:** 10.1017/S1368980025000321

**Published:** 2025-03-21

**Authors:** Liang Ma, Philip J Schluter

**Affiliations:** 1 Te Kaupeka Oranga | Faculty of Health, Te Whare Wānanga o Waitaha | University of Canterbury, Christchurch, New Zealand; 2 School of Clinical Medicine, Primary Care Clinical Unit, The University of Queensland, Brisbane, Australia

**Keywords:** Weight status, Weight misperception, Children and adolescents, Epidemiology, China

## Abstract

**Objective:**

Weight misperception has been reported as a common problem in high-income countries, but there is a paucity of high-quality empirical evidence in low- and middle-income countries, especially among children and adolescents. This study estimates the prevalence of weight misperception and investigates changes over time among children and adolescents in China, as well as identifies factors that may affect this weight misperception.

**Design:**

The China Health and Nutrition Survey, which is a repeated, representative cross-sectional study employing multistage random cluster processes.

**Setting:**

A Chinese national survey across fifteen provinces and municipal cities.

**Participants:**

Data from children and adolescents aged 6–16 years from six consecutive waves between 2000 and 2015 were included.

**Results:**

The final sample totalled 7110 children and adolescents. The overall prevalence of weight misperception was largely stable between 2000 and 2015 (range: 34·1–37·3 %). Sex and age groups were associated with weight misperception, with boys and younger participants more likely to misperceive their weight status. In addition, dieting and being physically active or inactive were associated with increased rates of weight misperception.

**Conclusions:**

Weight misperception is common among youth in China and is unequally shared with several subpopulations at increased risk. Researchers and health promoters are called to recognise weight misperception when addressing overweight and obesity countermeasures, and more tailored public health initiatives are warranted to more effectively reach those with weight misperceptions.

Overweight and obesity in childhood and adolescence are complex issues and are known to increase the likelihood of adverse social, economic and health consequences over the life course^(1)^. While these negative sequelae are well known, rates of overweight and obesity in children and adolescents continue to worsen across the globe. Between 1975 and 2016, the worldwide prevalence of obesity increased over eightfold among children and adolescents (i.e. from 0·7 to 5·6 % in girls and 0·9 to 7·8 % in boys)^(2)^. Although overweight and obesity rates among children and adolescents have plateaued in some high-income countries, they are now increasing in many low- and middle-income countries^(2,3)^. Consequently, an estimated 340 million children and adolescents were overweight or obese in 2016^(4)^. Overweight and obesity are largely preventable, but this, in part, relies on individual responsibility. Fundamental to this responsibility is having an accurate weight self-perception.

Like overweight and obesity itself, weight misperception (conceptualised as a discrepancy between measured and perceived weight status) is associated with a wide range of health problems, including eating disorders^(5–7)^, and psychological symptoms^(6,8)^. Among individuals within a normal weight range, those who perceive themselves as overweight are more likely to have unhealthy weight loss behaviours^(9)^. In contrast, overweight individuals who underestimate their weight status may have lower intention for healthy eating^(10)^, are less likely to control their weight^(11)^ and are associated with increased risk of some obesity-related problems such as CVD^(12)^.

Similar to overweight and obesity, weight misperception has been initially reported as a problem in high-income countries^(9,10,13,14)^. More recently, there is emerging evidence that this is also an issue within low- and middle-income countries^(15–17)^, including China, where mean BMI and obesity rates have increased steadily since the early 1980s^(18)^. Although a number of large nationwide prevention programmes targeting overweight and obesity have been implemented in China over recent years, their efficacy is likely to have been hampered by this weight misperception. Overweight and obese individuals who underestimate their weight status, thereby normalise their body size, are unlikely to consider their excess weight as a health problem, resulting in low intentions for weight loss.

Concurrently, China has witnessed dramatic shifts in cultural beliefs and body image ideals following the rapid economic growth in recent decades^(19,20)^. These shifts have exerted additional influence on citizens’ perception of their body image. Not surprisingly, a high proportion of Chinese children and adolescents now report body image dissatisfaction^(21)^. Further complicating matters, there is some evidence that weight misperception in China was found to be prevalent among children and adolescents^(17,22)^. In comparison with adults, children and adolescents are more vulnerable since they are experiencing a critical period in the life course, which encompasses elements of biological growth and major social role transitions^(23,24)^. As such, it is important to reliably understand the magnitude of weight misperception among youth in China and whether it is significantly worsening over time.

Extant literature focusing on weight misperception within low- and middle-income countries is rare and frequently limited to convenience sampling strategies, single cross-sectional designs and a lack of follow-up over a longer timeframe. Furthermore, understanding the factors that affect weight misperception is necessary for health professionals in developing tailored public health programmes aimed at addressing weight misperception and co-occurring outcomes. Prior findings from high-income countries and some middle-income countries indicated that weight misperception in adults is susceptible to a range of factors, which might vary by countries due to different participant characteristics^(10,25,26)^. Whether the influence of these factors on youth in China is open to conjecture due to its unique cultural and socio-economic characteristics, and thus, they warrant further investigation.

Using data collected from the repeatedly implemented China Health and Nutrition Survey (CHNS), this study aimed to estimate the prevalence of weight misperception among children and adolescents in China and investigate changes in this prevalence over time. The study also aimed to investigate and report on the demographic, family and sociocultural factors associated with weight misperception.

## Methods

### Study design

The CHNS is a repeated, nationally representative, cross-sectional study. It is an internationally collaborative project between the University of North Carolina at Chapel Hill and the Chinese Centers for Disease Control and Prevention and was designed to examine how the social and economic transformation of Chinese society affects a wide array of nutrition and health-related outcomes^(27)^. Initiated in 1989, the CHNS has been conducted eleven times, with the latest survey undertaken in 2015. However, information on body image has been collected since 2000. Therefore, six consecutive CHNS measurement waves were employed for the present study; the first measurement wave started in 2000, when both measured and perceived weight status were elicited, through to 2015.

### Participants

Detailed information on the CHNS study population, sample and quality control procedures appears in https://www.cpc.unc.edu/projects/china/about/design/sample. In brief, the study population is drawn from multiple districts (i.e. provinces and autonomous cities) of China, with variation in a wide range of socio-economic factors and other related health, nutritional and demographic measures. A multistage, random cluster process was used to draw the sample surveyed in each of the districts. Counties in the districts were stratified by income (low, middle and high), and a weighted sampling scheme was used to randomly select four counties in each district. Villages and townships within the counties and urban/suburban neighbourhoods within the cities were selected randomly. For the present study, children and adolescents aged between 6 years and 16 years deriving from the CHNS measurement waves of 2000, 2004, 2006, 2009, 2011 and 2015 were eligible for our analyses.

### Primary measure

Weight and height were measured by trained interviewers while the participants wore light clothing with shoes removed. Weight was measured to the nearest 0·1 kg using a calibrated scale. Height was measured using a fixed stadiometer and recorded to the nearest 0·1 cm. BMI was calculated by dividing weight in kilograms by the square of height in metres (kg/m^2^). In accordance with the WHO Child Growth Standards^(28,29)^, BMI-for-age Z-scores were selected as an indicator of measured weight status. Under this criterion, measured weight status was collapsed into three categories using the sd scores (sds): underweight (< −2 sds); normal weight (−2 sds to 1 sds); and overweight (> 1 sd)^(29)^. Children with BMI z-scores < –5 or > 5 were flagged as being biologically implausible and were excluded. Perceived weight status was measured by ‘Do you think you are now underweight, normal or overweight?’ Weight misperception was defined as discordance between the measured and perceived weight status classifications.

### Demographic, family, lifestyle and media-related variables

In addition to the primary variables, information about demographic, family, lifestyle and media-related variables was collected from the CHNS questionnaire. The choice of these variables that might produce an influence on weight misperception was based on the relevant literature^(24,30)^. Five indicators were selected as demographic variables, including sex, age group, locality, ethnicity and region of residence. Paternal and maternal presence were assessed as family variables. We considered physical activity, dieting, fast food consumption, snacking while watching TV and watching TV while eating meals as lifestyle variables. In addition, access to internet and bedroom TV were evaluated as media-related variables. A detailed description of the names, measures, response options and codings of these variables is presented within the online Supplementary Materials Table S1.

### Statistical analysis

Reporting of analyses was informed by the STROBE guidelines (www.strobe-statement.org). Frequencies, together with weighted percentages on participants’ demographic characteristics, were described by measurement waves. Weight misperception for the total observations was calculated, and trends in the prevalence were described.

The pattern of weight misperception rates over time was investigated using degree-2 fractional polynomial regression models from the set of powers (–2; –1; –0·5; 0; 0·5; 1; 2; 3)^(31)^. The best time polynomial specification was then used in all subsequent regression analyses. In addition to the overall rates, we stratified the sample by age group and sex to explore whether weight misperception was equally distributed between these subgroups.

The potential factors of weight misperception were explored through complete case multivariable analyses. As conventionally employed logistic regression models produce biased and inflated estimates when the outcome of interest is not rare, a modified Poisson regression approach (with log-link function and robust variance estimator) was used to estimate prevalence ratios (PR) directly^(32)^. A base model and adjusted models were employed. For the base model, only demographic variables (i.e. sex, age group, ethnicity, region of residence and locality) were analysed. A fully adjusted model was subsequently conducted. Rather than employing bivariable analyses to screen risk factors, all candidate variables were included in the adjusted model regardless of their statistical significance^(33)^. The area under the curve of the receiver operating characteristic (AUC) was used to assess this adjusted model’s predictive accuracy. In accordance with Hosmer and Lemeshow’s recommendations, an AUC of 0·5 indicates no discrimination, 0·7–0·8 is regarded as acceptable, 0·8–0·9 is regarded as excellent and more than 0·9 is regarded as outstanding^(34)^. Finally, in an effort to account for the missing data, sensitivity analyses were conducted on all regression models using chained equations multiple imputation (*M* = 50) methods^(35)^. All demographic and study variables were included within the multiple imputations. PR and associated 95 % CI were reported, and Wald’s type III *χ* ^2^ statistic was used to determine variable significance within these regression models. All the analyses were performed with Stata SE version 18.0 (StataCorp), with a two-sided *P* ≤ 0·05 considered significant.

## Results

### Participants

The final sample was composed of 1149 (16·1 %) participants from measurement wave 1 (2000), 1355 (19·1 %) from measurement wave 2 (2004), 1072 (15·1 %) from measurement wave 3 (2006), 1016 (14·3 %) from measurement wave 4 (2009), 1420 (20·0 %) from measurement wave 5 (2011) and 1098 (15·4 %) from measurement wave 6 (2015) and when combined totalled 7110 children and adolescents. Participants’ mean age was 11·0 years (range: 6–16 years), and 3393 (47·7 %) were female. Table 1 describes the participants’ demographic characteristics. Overall, 55·8 % (3966) were children aged 6–11 years, 14·7 % (1042) were identified as ethnic minority, 19·5 % (1390) were from North, 17·3 % (1232) from West, 30·0 % (2132) from East and 33·1 % (2356) were from Central China, and 63·3 % (4455) were living in rural areas.

**Table 1:** Participants’ demographic characteristics by measurement wave

	2000		2004		2006		2011		2014		2015		Total	
	*n* 1149		*n* 1355		*n* 1072		*n* 1016		*n* 1420		*n* 1098		*n* 7110	
	*n*	%	*n*	%	*n*	%	*n*	%	*n*	%	*n*	%	*n*	%
Age (6–11 years)	541	47·1	624	46·1	597	55·7	560	55·1	858	60·4	786	71·6	3966·	55·8
Sex (girls)	583	50·7	643	47·5	507	47·3	454	44·7	686	48·3	520	47·4	3393·	47·7
Ethnicity (minority)*	153	13·4	199	14·7	181	16·9	172	17·0	180	12·7	157	14·4	1042	14·7
Region of residence														
North	241	21·0	316	23·3	227	21·2	154	15·2	267	18·8	185	16·8	1390	19·5
West	147	12·8	227	16·8	169	15·8	186	18·3	304	21·4	199	18·1	1232	17·3
East	494	43·0	44·	32·8	317	29·6	304	29·9	285	20·1	288	26·2	2132	30·0
Central	267	23·2	368	27·2	359	33·5	372	36·6	564	39·7	426	38·8	2356	33·1
Locality (rural)^†^	741	68·4	882	65·4	703	65·6	676	66·5	779	54·9	674	61·4	4455	62·7

Note: *missing values for seven (2000), two (2009), four (2011) and nine (2015) participants; ^†^missing data for sixty-six (2000), six (2004) and one (2015) participants.

### Weight misperception: distribution of measured and perceived weight status

Valid measured and self-report perception data were available from all 7110 participants. Based on anthropometric measurements, 1011 (14·2 %) children and adolescents were classified as underweight, 5054 (71·1 %) as normal and 1045 (14·7 %) as overweight. For perceived weight status, 1445 (20·3 %), 4885 (68·7 %) and 780 (11·0 %) reported being underweight, normal and overweight, respectively. Table 2 presents the matched distribution of these measured and perceived weight status classifications. Overall, 2530 (35·6 %) participants misperceived their weight status. The majority of underweight (57·2 %) and overweight (62·3 %) participants misperceived their measured weight classification. In contrast, most individuals within the normal range (74·3 %) perceived themselves accurately.

**Table 2:** Participants’ distribution of measured and perceived weight status (*n* 7110)

Measured weight status	Perceived weight status			Total	Overall discrepancy (misperception)
	Underweight	Normal	Overweight		
	*n*%	*n*%	*n*%	*n*%	*n*%
Underweight	433 (42·8)	546 (54·0)	323 (·2)	1011 (14·2)	578 (57·2)
Normal	947 (18·7)	3753 (74·3)	354 (7·0)	5054 (71·1)	1301 (25·7)
Overweight	65 (6·2)	586 (6·2)	394 (37·7)	1045 (14·7)	651 (62·3)
Total	1445 (20·3)	4885 (68·7)	780 (11·0)	7110 (100)	2530 (35·6)

### Prevalence of weight misperception

**Fig. 1:**
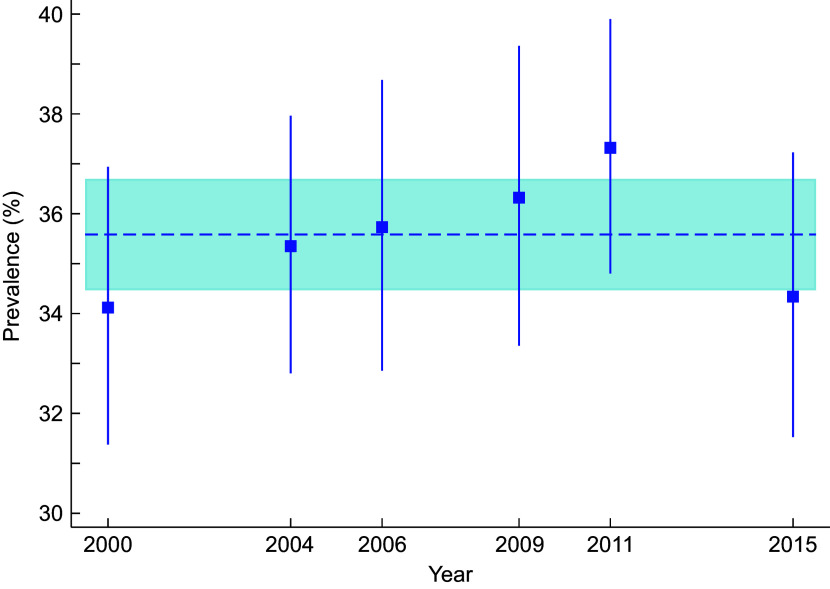
Estimated prevalence of weight misperception (squares) and associated 95 % CI (solid lines) by year, together with the overall estimated mean prevalence (dashed line) and associated 95 % CI (shaded area).

The overall prevalence of weight misperception was 35·6 %, ranging from 34·1 % in 2000 to 37·3 % in 2011. Patterns in weight misperception over measurement waves were investigated using degree-2 fractional modified Poisson regression models. There was no evidence for first- or second-order relationships, implying that the prevalence of weight misperception was largely constant over the study period. Figure 1 depicts the estimated prevalence of weight misperception and associated 95 % CI by year, together with the overall constant-only estimated mean prevalence and associated 95 % CI.

The potentially differential influence of age group and sex on weight misperception was next investigated. In modified Poisson regression analyses, a significant difference in misperception was found between sexes (*P* = 0·02) and age groups (*P* < 0·001), but no evidence for an age group × sex interaction existed (*P* = 0·52). In these analyses, the estimated prevalence ratio (PR) for male misperception was 1·08 (95 % CI: 1·01, 1·15) compared with females and 1·24 (95 % CI: 1·16, 1·32) for children compared with adolescents. The estimated prevalence of weight misperception was 40·3 % (95 % CI: 38·4 %, 42·2 %) for male children, 37·2 % (95 % CI: 35·3 %, 39·3 %) for female children, 32·7 % (95 % CI: 30·8 %, 34·7 %) for male adolescents and 30·2 % (95 % CI: 28·4 %, 32·2 %) for female adolescents.

### Potential factors affecting weight misperception

In the 2000 measurement wave, two variables of interest (access to internet and bedroom TV) were not collected, and thus, this secondary analysis was limited to the 2004–2015 measurement waves (*n* 5961). The distribution of weight misperception for demographic and potentially confounding variables for this subsample appears in Table 3.

**Table 3:** Distribution of weight misperception for demographic and potentially confounding variables, together with estimated proportional odds (PR) and associated 95 % CI for a base model including demographic variables (*n* 5939), a complete case multivariable models (*n* 3292) and multivariate multiple imputed model (*n* 5961) using modified Poisson regression analyses

		Misperception		Base model		Multivariable (complete case)		Multivariable (multiple imputed)	
	*n*	*n*	%	PR	95 % CI	PR	95 % CI	PR	95 % CI
*Sex*									
Male	3151	1174	37·3	1·08	1·01, 1·16	1·08	0·99, 1·18	1·10	1·02, 1·17
Female	2810	964	34·3	1	reference	1	reference	1	reference
Age group									
Child (6–11 years)	3425	1342	39·2	1·25	1·16, 1·34	1·16	1·05, 1·29	1·24	1·15, 1·34
Adolescent (12–16 years)	2536	796	31·4	1	reference	1	reference	1	reference
Ethnicity									
Han Chinese	5057	1827	36·1	1	reference	1	reference	1	reference
Minority	889	307	34·5	0·95	0·86, 1·05	0·90	0·78, 1·04	0·95	0·86, 1·06
Region of residence									
North	1149	407	35·4	1	reference	1	reference	1	reference
West	1085	381	35·1	0·97	0·87, 1·09	1·02	0·87, 1·20	1·00	0·89, 1·12
East	1638	601	36·7	1·01	0·91, 1·12	1·10	0·95, 1·27	1·03	0·93, 1·14
Central	2089	749	35·9	0·99	0·90, 1·09	1·03	0·89, 1·18	1·00	0·90, 1·11
Locality									
Urban	2240	788	35·2	1	reference	1	reference	1	reference
Rural	3714	1346	36·2	1·02	0·95, 1·09	1·00	0·90, 1·11	1·00	0·93, 1·09
Paternal presence									
Yes	4841	1701	35·1			1	reference	1	reference
No	1118	436	39·0			1·04	0·90, 1·20	1·09	0·99, 1·20
Maternal presence									
Yes	5124	1818	35·5			1	reference	1	reference
No	836	320	38·3			1·01	0·86, 1·18	1·01	0·91, 1·14
Physical activity									
Too little	1674	665	39·7			1·23	1·11, 1·35	1·21	1·12, 1·31
About right	3526	1162	33·0			1	reference	1	reference
Too much	207	104	50·2			1·56	1·30, 1·88	1·48	1·28, 1·71
Dieting									
No	5412	1908	35·3			1	reference	1	reference
Yes	452	196	43·4			1·11	0·95, 1·30	1·20	1·07, 1·35
Fast food consumption									
< 1–2 times/week	3188	1155	36·2			1	reference	1	reference
1–2 times/week	876	310	35·4			0·98	0·86, 1·11	0·98	0·88, 1·09
≥ 3 times/week	666	230	34·5			0·92	0·79, 1·06	0·96	0·84, 1·09
Snacking while watching TV									
Seldom	2883	1025	35·6			1	reference	1	reference
Sometimes	1117	407	36·4			1·00	0·89, 1·12	1·00	0·91, 1·10
Often	425	154	36·2			0·93	0·79, 1·10	1·00	0·87, 1·15
Eat meals while watching TV									
Seldom	2319	807	34·8			1	reference	1	reference
Sometimes	1129	421	37·3			1·08	0·96, 1·21	1·07	0·97, 1·17
Often	982	360	36·7			1·02	0·91, 1·15	1·03	0·93, 1·14
Access to internet									
No	3966	1464	36·9			1	reference	1	reference
Yes	1881	633	33·7			0·94	0·85, 1·05	0·96	0·88, 1·04
Bedroom TV									
No	4798	1709	35·6			1	reference	1	reference
Yes	946	347	36·7			0·98	0·86, 1·10	1·01	0·92, 1·11

For the base model (*n* 5939; 99·6 %), the effects of demographic variables (i.e. sex, age group, ethnicity, region of residence and locality) on weight misperception (Table 3) were initially investigated. In this model, age group was significant (*P* < 0·001), with younger participants more likely to misperceive their weight status than adolescents. Sex was also significant (*P* = 0·02), with boys having a higher PR of weight misperception compared with girls. In contrast, ethnicity, locality and region of residence were all non-significant.

Following the base model, a complete case multivariable model was undertaken (*n* 3292; 55·2 %) investigating the effects of family, lifestyle and media-related variables on weight misperception; see Table 3. Among the considered potential factors affecting weight misperception, the median pairwise correlation was 0·01, with the highest between paternal and maternal presence (*r* = 0·56). In this model, age group remained significant (*P* = 0·005), as did physical activity (*P* < 0·001), but all other considered variables were non-significant. As before, weight misperception among children had a higher PR than among adolescents. Interestingly, both too little and too much self-reported physical activity were also associated with higher PR estimates of weight misperception. The estimated AUC for this model was 0·58 (95 % CI: 0·56, 0·60), which is not considered predictive acceptable, suggesting that other important unmeasured confounders exist^(34)^. However, there was no evidence that the model assumptions were violated (deviance goodness-of-fit *P* > 0·99).

**Fig. 2:**
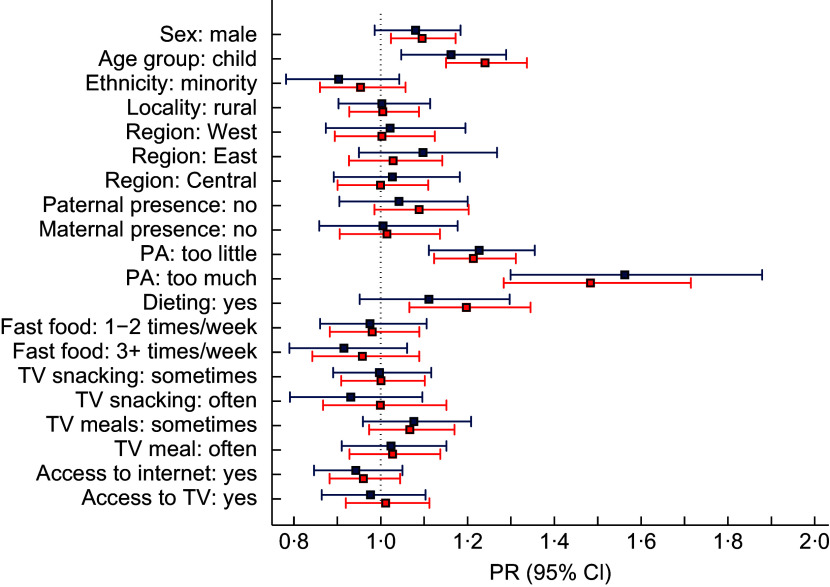
Estimated prevalence ratio (PR) and associated 95 % CI of factors associated with weight misperception derived from complete case (blue; *n* 3292) and multiple imputed (red; *n* 5961) analyses from 2004 to 2015.

Given that only 55·2 % of the subsample was utilised in the complete case multivariable analysis, a sensitivity analysis was undertaken using multiple imputed data (*M* = 50). Table 3 and Fig. 2 present estimated PR and associated 95 % CI of the factors associated with weight misperception derived from both complete case (*n* 3292) and multiple imputed (*n* 5961) analyses. In the multiple imputed multivariable modified Poisson regression analysis, age group remained significant (*P* < 0·001), as did sex (*P* = 0·009), physical activity (*P* < 0·001) and whether participants were on a diet (*P* = 0·002). The direction of the effect sizes for age group, sex and physical activity was the same as described earlier. Being on a diet was, however, a new addition to this suite of significant variables – with those reporting dieting having an estimated adjusted PR of weight misperception 1·20 (95 % CI: 1·07, 1·35) higher than their counterparts not dieting. When compared with the complete case PR estimates, the multiple imputed estimates generally exhibited modest shrinkage towards the null, with age group and dieting being notable exceptions; see Fig. 2.

## Discussion

Weight misperception among youth in China was observed to be common and largely constant between 2000 and 2015. Our prevalence estimates are somewhat smaller relative to those (range: 34·5–56·6 %) found in other studies targeting children and adolescents in China^(7,17,22)^, likely due to convenience sampling bias within these studies. When comparing between countries, our prevalence estimates are comparable to reports from some other low- and middle-income countries^(16)^ and high-income countries^(36)^. Overall, these findings reinforce the view that weight misperception is a common public health challenge for youth in China. Since weight misperception can limit the effectiveness of public health initiatives aimed at reducing excess weight in this population, implementing prevention programmes recognising this discordance needs to be developed or tailored accordingly.

Both age group and sex were associated with weight misperception among children and adolescents. The prevalence decreased as age groups increased, a finding consistent with the literature^(14)^. One plausible interpretation for this finding is the higher cognitive ability for older than younger age groups. In comparison with girls, boys in this study seem more likely to misperceive their weight status. It is possible that the pursuit of muscularity in boys may lead them to underestimate their weight status despite a high BMI^(37,38)^. It is also possible that girls have greater awareness of their weight status since they may be relatively more concerned about their body image. Owing to cultural norms and societal pressures from mass and social media, girls tend to demonstrate greater anti-fat attitudes and more positive views of thinness compared with boys^(39)^. These sex differences are supported by two additional studies conducted in Chinese children and adolescents^(17,22)^. However, studies in other similarly aged populations in different countries have also observed the reverse, with more females than males misperceiving their weight^(8,40)^.

Another priority of this study was to explore potential factors that affect weight misperception among children and adolescents. In addition to the frequently evaluated demographic factors, a range of family, lifestyle and media-related factors were considered in our analyses, resulting in some significant findings. These findings were largely reliable even though a set of different models were employed, underlining that the factors identified in our analyses might be significant predictors of weight misperception among children and adolescents. Such information is important since it would assist health professionals in identifying subgroups in which weight misperception is most prevalent, which could guide future programme development and implementation efforts targeting this population.

A finding of interest was that healthy eating and recommended physical activity levels reduced the risk of weight misperception among children and adolescents. Our results suggest that children and adolescents who are on a diet to lose/gain weight are more likely to misperceive their weight status. In support, several other studies have demonstrated the relationship between disordered eating behaviours and weight misperception among children and adolescents^(41–43)^. In addition, compared with the appropriate physical activity levels, both a higher and a lower level of physical activity were associated with a greater risk of weight misperception. It is possible that physically active individuals are more likely to underestimate their weight status and perhaps have a higher ratio of muscle to fat. In contrast, physically inactive individuals, especially those who have a lower ratio of muscle to fat, may overestimate their weight status. In line with this, several studies have confirmed that weight misperception is more prevalent among those physically active individuals^(10,44)^, and some other studies found weight misperception to be associated with physical inactivity^(26,41,45,46)^. Overall, these findings support that living a healthy nutritional and physical lifestyle may assist in reducing weight misperception among children and adolescents.

In addition to the aforementioned factors, it is noteworthy that several factors were not significantly associated with weight misperception. Although it might be interpreted that these factors do not affect weight misperception, some of these null findings warrant further investigation. For instance, since parents are critical in the socialisation of young children, their weight-related attitudes ought to play an important role in the development of weight bias in their children. This has been supported by a range of studies, including Rich and colleagues, who found that parental body dissatisfaction was associated with attributing negative traits to overweight and positive traits to thinness^(47)^. Spiel and colleagues found that the father was crucial in the transmission of weight bias among children, especially boys^(48)^. Other researchers showed that parents can positively or negatively influence their children’s weight attitudes through the modelling of weight and dieting behaviours as well as their reinforcement through comments^(49,50)^. In contrast, neither paternal presence nor maternal presence was associated with children’s weight misperception in our analyses; those living with a father/mother showed similar risk to others not living in this arrangement. Our results reveal that although parents exert important influence on their children, solely living with a father/mother does not significantly attenuate weight misperception. Future studies are needed to evaluate the effect of other parental factors on weight misperception.

### Strengths and limitations

While this study encompasses important strengths, including the robust survey design, the large representative sample (i.e. multistage, random cluster sampling was applied, resulting in a wide spread of participants across China) and the repeated follow-up measures, which together have contributed to producing reliable and robust estimates, several limitations should be noted. Despite measured BMI z-scores, data collected for perceived weight status and other variables were primarily self-reported, which may lead to recall bias and response bias. Another limitation is that the repeated cross-sectional design of this study provided only correlational instead of causal relations. This study is also limited to exploring the factors that may affect weight misperception, since other non-specified factors were not analysed. In addition, while the majority of geographical regions were consistent across the study period, three megacities joined this cohort since 2011, and three more provinces joined since 2015, contributing to potential sampling bias. Furthermore, information on body image was only collected from 2000, limiting our ability to identify the trend over a longer timeframe. Meanwhile, much has happened since the latest CHNS (held over 2015), including the outbreak of COVID-19, and so more recent patterns may be different.

### Conclusions

Using a repeated cross-sectional design utilising data from six representative CHNS surveys, we assessed the prevalence and correlates of weight misperception among Chinese children and adolescents. The findings reveal that weight misperception is common and largely constant among youth in China. Weight misperception is also unequally distributed and more prevalent within a range of subpopulations (e.g. boys, young children, those on a diet and those being physically active or inactive). Researchers and health promoters are called to recognise weight misperception when addressing overweight and obesity countermeasures, and more tailored public health initiatives are warranted to more effectively reach those at risk.

## Supporting information

Ma and Schluter supplementary material Ma and Schluter supplementary material

## Supplementary material

For supplementary material accompanying this paper, visit https://doi.org/10.1017/S1368980025000321

## References

[1] Jebeile H , Kelly AS , O’Malley G et al. (2022) Obesity in children and adolescents: epidemiology, causes, assessment, and management. Lancet Diabetes Endocrinol 10, 351–365. 35248172 10.1016/S2213-8587(22)00047-X PMC9831747

[2] NCD Risk Factor Collaboration (NCD-RisC) (2017) Worldwide trends in body-mass index, underweight, overweight, and obesity from 1975 to 2016: a pooled analysis of 2416 population-based measurement studies in 128·9 million children, adolescents, and adults. Lancet 390, 2627–2642. 29029897 10.1016/S0140-6736(17)32129-3 PMC5735219

[3] Lobstein T , Jackson-Leach R , Moodie ML et al. (2015) Child and adolescent obesity: part of a bigger picture. Lancet 385, 2510–2520. 25703114 10.1016/S0140-6736(14)61746-3 PMC4594797

[4] World Health Organization (2021) Obesity and Overweight. https://www.who.int/news-room/fact-sheets/detail/obesity-and-overweight (accessed 19 April 2024).

[5] Hazzard VM , Hahn SL & Sonneville KR (2017) Weight misperception and disordered weight control behaviors among U.S. high school students with overweight and obesity: associations and trends, 1999–2013. Eat Behav 26, 189–195. 28734231 10.1016/j.eatbeh.2017.07.001

[6] Isomaa R , Isomaa AL , Marttunen M et al. (2011) Longitudinal concomitants of incorrect weight perception in female and male adolescents. Body Image 8, 58–63. 21147053 10.1016/j.bodyim.2010.11.005

[7] Yan H , Wu Y , Oniffrey T et al. (2018) Body weight misperception and its association with unhealthy eating behaviors among adolescents in China. Int J Environ Res Public Health 15, 936. 29738429 10.3390/ijerph15050936 PMC5981975

[8] Elia C , Karamanos A , Silva MJ et al. (2020) Weight misperception and psychological symptoms from adolescence to young adulthood: longitudinal study of an ethnically diverse UK cohort. BMC Public Health 20, 712. 32423390 10.1186/s12889-020-08823-1 PMC7236343

[9] Liechty JM (2010) Body image distortion and three types of weight loss behaviors among nonoverweight girls in the United States. J Adolesc Health 47, 176–182. 20638010 10.1016/j.jadohealth.2010.01.004

[10] Matthiessen J , Biltoft-Jensen A , Fagt S et al. (2014) Misperception of body weight among overweight Danish adults: trends from 1995 to 2008. Public Health Nutr 17, 1439–1446. 23735172 10.1017/S1368980013001444 PMC10282460

[11] Sonneville KR , Thurston IB , Milliren CE et al. (2016) Weight misperception among young adults with overweight/obesity associated with disordered eating behaviors. Int J Eat Disord 49, 937–946. 27218865 10.1002/eat.22565 PMC5064910

[12] Lee K (2023) Moderation of weight misperception on the associations between obesity indices and estimated cardiovascular disease risk. Int J Behav Med 30, 89–96. 35257308 10.1007/s12529-022-10073-x

[13] Marques-Vidal P , Melich-Cerveira J , Marcelino G et al. (2011) High- and persistent- body-weight misperception levels in overweight and obese Swiss adults, 1997–2007. Int J Obes (Lond) 35, 1549–1550. 21266952 10.1038/ijo.2010.285

[14] Salcedo V , Gutiérrez-Fisac JL , Guallar-Castillón P et al. (2010) Trends in overweight and misperceived overweight in Spain from 1987 to 2007. Int J Obes (Lond) 34, 1759–1765. 20498661 10.1038/ijo.2010.96

[15] Angoorani P , Heshmat R , Ejtahed HS et al. (2017) Body weight misperception and health-related factors among Iranian children and adolescents: the CASPIAN-V study. J Pediatr Endocrinol Metab 30, 1033–1040. 28888091 10.1515/jpem-2017-0149

[16] da Silva SU , Gonçalves VSS , Barufaldi LA et al. (2022) Weight misperception and substance use: Brazilian Study of Cardiovascular Risks in Adolescents (ERICA). BMC Public Health 22, 1850. 36192717 10.1186/s12889-022-14267-6 PMC9531377

[17] Qin TT , Xiong HG , Yan MM et al. (2019) Body weight misperception and weight disorders among Chinese children and adolescents: a latent class analysis. Curr Med Sci 39, 852–862. 31612407 10.1007/s11596-019-2116-1

[18] Wang L , Zhou B , Zhao Z et al. (2021) Body-mass index and obesity in urban and rural China: findings from consecutive nationally representative surveys during 2004–2018. Lancet 398, 53–63. 34217401 10.1016/S0140-6736(21)00798-4 PMC7617101

[19] Gao L , Ma L , Xue H et al. (2020) A 3-year longitudinal study of effects of parental perception of children’s ideal body image on child weight change: the Childhood Obesity Study in China mega-cities. Prev Med 132, 105971. 31899255 10.1016/j.ypmed.2019.105971 PMC7024657

[20] Zhang L , Qian H & Fu H (2018) To be thin but not healthy - the body-image dilemma may affect health among female university students in China. PLoS One 13, e0205282. 30304026 10.1371/journal.pone.0205282 PMC6179281

[21] Min J , Yan AF , Wang VHC et al. (2018) Obesity, body image, and its impact on children’s eating and exercise behaviors in China: a nationwide longitudinal study. Prev Med 106, 101–106. 29066373 10.1016/j.ypmed.2017.10.024 PMC5962018

[22] Zhao M , Zhang M , Zhou X et al. (2012) Weight misperception and its barriers to keep health weight in Chinese children. Acta Paediatr 101, e550–556. 23025796 10.1111/apa.12011

[23] Sawyer SM , Azzopardi PS , Wickremarathne D et al. (2018) The age of adolescence. Lancet Child Adolesc Health 2, 223–228. 30169257 10.1016/S2352-4642(18)30022-1

[24] Viner RM , Ozer EM , Denny S et al. (2012) Adolescence and the social determinants of health. Lancet 379, 1641–1652. 22538179 10.1016/S0140-6736(12)60149-4

[25] Andrade FCD , Raffaelli M , Teran-Garcia M et al. (2012) Weight status misperception among Mexican young adults. Body Image 9, 184–188. 22104126 10.1016/j.bodyim.2011.10.006

[26] Kye SY & Park K (2021) Gender differences in factors associated with body weight misperception. Public Health Nutr 24, 2483–2495. 32981555 10.1017/S1368980020003262 PMC10195502

[27] Zhang B , Zhai FY , Du SF et al. (2014) The China Health and Nutrition Survey, 1989–2011. Obes Rev 15, 2–7. 10.1111/obr.12119 PMC3869031 24341753

[28] Bloem M (2007) The 2006 WHO child growth standards. BMJ 334, 705–706. 17413142 10.1136/bmj.39155.658843.BE PMC1847861

[29] de Onis M , Onyango AW , Borghi E et al. (2007) Development of a WHO growth reference for school-aged children and adolescents. Bull World Health Organ 85, 660–667. 18026621 10.2471/BLT.07.043497 PMC2636412

[30] Pan XF , Wang L & Pan A (2021) Epidemiology and determinants of obesity in China. Lancet Diabetes Endocrinol 9, 373–392. 34022156 10.1016/S2213-8587(21)00045-0

[31] Royston P & Sauerbrei W (2008) Multivariable model-building: a pragmatic approach to regression analysis based on fractional polynomials for modelling continuous variables. Gen Inf 65, 989–990.

[32] Zou G (2004) A modified Poisson regression approach to prospective studies with binary data. Am J Epidemiol 159, 702–706. 15033648 10.1093/aje/kwh090

[33] Sun GW , Shook TL & Kay GL (1996) Inappropriate use of bivariable analysis to screen risk factors for use in multivariable analysis. J Clin Epidemiol 49, 907–916. 8699212 10.1016/0895-4356(96)00025-x

[34] Hosmer DW & Lemeshow S (2000) *Applied Logistic Regression*, 2nd ed. New York: Wiley.

[35] StataCorp (2023) *Stata Multiple-Imputation Reference Manual: Release 18*. College Station, TX: StataCorp LLC.

[36] Beech BM , Bruce MA , Cohen-Winans S et al. (2021) Weight misperception among African American adolescents: the Jackson Heart KIDS Pilot Study. Ethn Dis 31, 461–468. 34295134 10.18865/ed.31.3.461 PMC8288478

[37] Ricciardelli LA & McCabe MP (2004) A biopsychosocial model of disordered eating and the pursuit of muscularity in adolescent boys. Psychol Bull 130, 179–205. 14979769 10.1037/0033-2909.130.2.179

[38] Rodgers RF , Ganchou C , Franko DL et al. (2012) Drive for muscularity and disordered eating among French adolescent boys: a sociocultural model. Body Image 9, 318–323. 22494958 10.1016/j.bodyim.2012.03.002

[39] Holub SC (2008) Individual differences in the anti-fat attitudes of preschool-children: the importance of perceived body size. Body Image 5, 317–321. 18586582 10.1016/j.bodyim.2008.03.003

[40] San Martini MC , de Assumpção D , Barros MBA et al. (2021) Weight self-perception in adolescents: evidence from a population-based study. Public Health Nutr 24, 1648–1656. 33648614 10.1017/S1368980021000690 PMC10195491

[41] Dues K , Kandiah J , Khubchandani J et al. (2020) Adolescent body weight perception: association with diet and physical activity behaviors. J Sch Nurs 36, 339–347. 30674226 10.1177/1059840518824386

[42] Ibrahim C , El-Kamary SS , Bailey J et al. (2014) Inaccurate weight perception is associated with extreme weight-management practices in U.S. high school students. J Pediatr Gastroenterol Nutr 58, 368–375. 24172585 10.1097/MPG.0000000000000231 PMC3982798

[43] Lim H , Lee HJ , Park S et al. (2014) Weight misperception and its association with dieting methods and eating behaviors in South Korean adolescents. Nutr Res Pract 8, 213–219. 24741407 10.4162/nrp.2014.8.2.213 PMC3988512

[44] Hahn SL , Borton KA & Sonneville KR (2018) Cross-sectional associations between weight-related health behaviors and weight misperception among U.S. adolescents with overweight/obesity. BMC Public Health 18, 514. 29669539 10.1186/s12889-018-5394-9 PMC5907388

[45] Duncan DT , Wolin KY , Scharoun-Lee M et al. (2011) Does perception equal reality? Weight misperception in relation to weight-related attitudes and behaviors among overweight and obese US adults. Int J Behav Nutr Phys Act 8, 20. 21426567 10.1186/1479-5868-8-20 PMC3073863

[46] Murillo R , Ali SA , Carmack C et al. (2016) Activity and weight misperception among overweight and obese US adults. Am J Health Behav 40, 12–20. 26685809 10.5993/AJHB.40.1.2

[47] Rich SS , Essery EV , Sanborn CF et al. (2008) Predictors of body size stigmatization in Hispanic preschool children. Obesity (Silver Spring) 16, S11–17. 10.1038/oby.2008.446 18978757

[48] Spiel EC , Rodgers RF , Paxton SJ et al. (2016) ‘He’s got his father’s bias’: parental influence on weight bias in young children. Br J Dev Psychol 34, 198–211. 26666696 10.1111/bjdp.12123

[49] Ricciardelli LA & McCabe MP (2001) Children’s body image concerns and eating disturbance: a review of the literature. Clin Psychol Rev 21, 325–344. 11288604 10.1016/s0272-7358(99)00051-3

[50] Rodgers R & Chabrol H (2009) Parental attitudes, body image disturbance and disordered eating amongst adolescents and young adults: a review. Eur Eat Disord Rev 17, 137–151. 19130467 10.1002/erv.907

