# Supplementary material for: Weight misperception among Chinese children and adolescents: evidence from the repeated China Health and Nutrition Survey
Source: Public Health Nutr. 2025 Mar 21;28(1):e62. doi: 10.1017/S1368980025000321 (PMC12086734; doi:10.1017/S1368980025000321)
Supplement: Ma and Schluter supplementary material [file S1368980025000321sup001.docx]

**Table S1.** Names, measures, response options, and codings of the study variables.

| **Variables** | **Measure** | **Responses options** | **Coding** |
| --- | --- | --- | --- |
| **Demographic variables** |  |  |  |
| *Gender* | What gender is the participant? | Boy, girl | 1=boy, 2=girl |
| *Age* | How old is the participant? | Age in years | 0=children (ages 5-11), 1=adolescents (ages 12-16) |
| *Locality* | To which type of household registration does the participant belong? | Urban, rural | 1=urban, 2=rural |
| *Ethnic minority* | What ethnicity is the participant? | Han, otherwise | 1=Han, 2=otherwise |
| *Region of residence* | Which province does the participant come from? | Beijing, Liaoning, Heilongjiang, Guangxi, Guizhou, Chongqing, Shanghai, Jiangsu, Shandong, Henan, Hubei, Hunan | 1=North (Beijing, Liaoning, Heilongjiang), 2=West (Guangxi, Guizhou, Chongqing), 3=East (Shanghai, Jiangsu, Shandong), 4=Central (Henan, Hubei, Hunan) |
| **Family variables** |  |  |  |
| *Paternal presence* | Does your father live in this household? | Yes, no | 0=no, 1=yes |
| *Maternal presence* | Does your mother live in this household? | Yes, no | 0=no, 1=yes |
| **Lifestyle variables** |  |  |  |
| *Physical activity* | Do you think you have too little, just the right amount, or too much physical activity? | Too little, just the right amount, too much, unknown | 1=too little, 2=just the right amount, 3=too much |
| *Dieting* | Were you on a diet in the last year? | No, yes, on a diet to lose weight, yes, on a diet to gain weight, unknown | 0=no, 1=yes (on a diet to lose weight, on a diet to gain weight) |
| *Fast food consumption* | During the past 3 months, how many times have you eaten at a Western fast food restaurant, such as McDonald’s or Kentucky Fried Chicken? |  | 1=1-2 times/week, 2=3+times/week |
| *Snacking while watching TV* | Do you eat snacks while watching TV? | Very seldom (< 1 times/month), seldom (1-3 times/month), sometimes (1-2 times/week), often (3-4 times/week), very often (> 5 times/week), unknown | 1=seldom (very seldom, seldom), 2=sometimes, 3=often (often, very often) |
| *Eat meals while watching TV* | Do you watch TV when you are eating a meal? | Very seldom (< 1 times/month), seldom (1-3 times/month), sometimes (1-2 times/week), often (3-4 times/week), very often (> 5 times/week), unknown | 1=seldom (very seldom, seldom), 2=sometimes, 3=often (often, very often) |
| **Media-related variables** |  |  |  |
| *Access to internet* | Can you access to the internet? | Yes, no | 0=no, 1=yes |
| *TV-bedroom* | Do you have a TV (in working order) in your bedroom? | Yes, no | 0=no, 1=yes |
